# Adapting Policy Guidelines for Spine Surgeries During COVID-19 Pandemic in View of Evolving Evidences: An Early Experience From a Tertiary Care Teaching Hospital

**DOI:** 10.7759/cureus.9147

**Published:** 2020-07-11

**Authors:** Virendra Verma, Manoj Nagar, Vaibhav Jain, John A Santoshi, Manish Dwivedi, Prateek Behera, Rajkumar Selvanayagam, Dharm Pal, Kuldeep Singh

**Affiliations:** 1 Orthopaedics, All India Institute of Medical Sciences, Bhopal, IND

**Keywords:** covid-19, novel coronavirus, corona virus, emergency surgery, spinal surgery, trauma, spinal tuberculosis, vertebral fracture, covid-19 india

## Abstract

Introduction

The recent novel coronavirus disease 2019 (COVID-19) pandemic has brought the world to a standstill. This outbreak not only affected healthcare systems but the resultant economic losses were also enormous. COVID-19 has demanded that the health care systems globally evolve, develop new strategies, identify new models of functioning, and at times, fall back on the old conservative methods of orthopedic care to decrease the risk of disease transmission. Although, the majority of hospitals are refraining from performing elective surgeries, emergent and urgent procedures cannot be delayed. Various strategies have been developed at the institute level to reduce the risk of infection transmission among the theatre team from an unsuspected patient (asymptomatic and presymptomatic) during the perioperative period.

Material and methods

The present study is a part of an ongoing project which is being conducted in a tertiary level hospital after obtaining research review board approval. All patients admitted either for vertebral fracture or spinal cord compression from February 2020 to May 2020 were included. The present study included 13 patients (nine males and four females) with an average age of 35.4 years The oldest patient was of 63 years which is considered a risk factor for developing severe COVID-19 infection.

Results

Eight patients (61.5%) presented with spinal cord injury (SCI) due to vertebral fracture with fall from height (87.5%) as the most common etiology. Among the traumatic SCI patients, six (75%) were managed surgically with posterior decompression and instrumented fusion with pedicle screws while two patients (25%) were managed conservatively. There were four patients (30.8%) of tuberculosis of the spine of whom two (50%) were managed with posterior decompression, debridement, and stabilization with pedicle screws, samples for culture, biopsy, and cartridge-based nucleic acid amplification test (CBNAAT) were collected during the procedure; for the remaining two patients (50%), a trans-pedicular biopsy was performed to confirm the diagnosis for initiation of anti-tubercular therapy. Prolapsed intervertebral disc causing cauda equina syndrome was the reason for emergency surgery in one patient (7.7%). COVID-19 severe acute respiratory syndrome coronavirus 2 (SARS-CoV-2) reverse transcription-polymerase chain reaction (RT-PCR) test was performed in four patients (30.8%), in whom the most common symptom was fever (two patients (50%)). These patients were residents of high prevalence area for COVID-19 infection. Sore throat (25%), fatigue (25%), and low oxygen saturation (25%) were present in one patient which prompted us to get the COVID-19 test. All patients were reported negative for COVID-19.

Conclusion

The structural organization and the management protocol we describe allowed us to reduce infection risk and ultimately hospital stay, thereby maximizing the already stretched available medical resources. These precautions helped us to reduce transmission and exposure to COVID-19 in health care workers (HCW) and patients in our institute. The aim of this article is that our early experience can be of value to the medical communities that will soon be in a similar situation.

## Introduction

The recent novel coronavirus disease 2019 (COVID-19) pandemic has brought the world to a standstill. This outbreak not only affected the healthcare systems but the resultant economic losses were enormous. COVID-19 has demanded that the health care systems globally evolve, develop new strategies, identify new models of functioning, and at times, fall back on the old conservative methods of orthopedic care to decrease the risk of disease transmission.

The usual incubation period of the disease is 5-14 days but with reported outliers up to 24 days [1]. It is not just the contagiousness of the COVID-19 but the alarming fatality as well (approximately 3.6%), which far exceeds the other severe acute respiratory syndrome (SARS) viruses [1]. Although the recent World Health Organization (WHO) report suggests that the asymptomatic cases are less likely to transmit the virus, this is debatable with conflicting evidence. Moreover, the presymptomatic cases are as likely to transmit the virus [2,3].

Infection among health care workers (HCWs) is further straining the already overburdened workforce due to a sudden spike in the caseload. Although, the majority of hospitals are refraining from performing elective surgeries, emergent and urgent procedures cannot be delayed. Various strategies have been developed at the institute level to reduce the risk of infection transmission among the theatre team from an unsuspected patient (asymptomatic and presymptomatic) during the perioperative period [2,3].

India has rapidly surged its capacity of reverse transcription-polymerase chain reaction (RT-PCR) testing for severe acute respiratory syndrome coronavirus 2 (SARS-CoV-2) but with its 1.3 billion population and a rapidly increasing infection rate, a selective testing approach was adopted initially. The policy regarding testing in India formulated by the Indian council of medical research (ICMR) has been revised with evolving evidence regarding the spread of infection [4]. 

Since testing could not be performed in all patients in need of surgical intervention due to time or resource constrain, nor an initial negative test can surely rule out infection, a “consider all positive” approach was adopted by the authors' institute to manage patients requiring emergent or urgent care including the available recommendations regarding reducing the risk of exposure to the surgical team during the perioperative period,

We developed a “caution at each step” model in an effort to minimize the risk of exposure to the theatre team from an unsuspected patient. In this study, we elaborate on this model in detail along with our experience of its use in emergency spine surgery cases.

## Materials and methods

The patients participating are also part of an ongoing prospective study on spine injuries at the authors' institute for which institutional ethics committee approval has been obtained. All patients admitted either for vertebral fracture or spinal cord compression from February 2020 to May 2020 were included. The spine conditions included were spinal cord injury (SCI), epidural abscess, epidural hematoma, close reduction of cervical facet dislocation, cauda equine syndrome, surgical spine tumor with spinal cord compression (Table 1). No COVID-19 patients were treated. The neurological status was assessed using the American Spinal Injury Association (ASIA) impairment scale as it can be used for traumatic [5] as well as non-traumatic SCI [6]. Thoracolumbar injury classification and severity score (TLICS) based on the morphology of the injury, the integrity of the posterior ligamentous complex, and neurological status of the patient was performed in which points are assigned for each category and the final total points suggest the possible treatment option for thoracolumbar vertebral fractures [7].

**Table 1:** Inclusion and exclusion criterias

A) Inclusion Criteria (Indications for emergency surgery)
1) Spinal Cord injury due to verbral fracture
2) Epidural Abscess
3) Epidural Haematoma
4) Surgical Spine tumour with Spinal Cord compression with progressive neurological deficit
5) Cauda equina syndrome
6) Infective pathology of spine (tubercular spondylodiscitis,bacterial spondylodiscitis)
B) Exclusion Criteria (Surgeries which can be delayed)
1) Surgical lumbar disk herniation with radiculopathy
2) Spondylolisthesis
3) Surgical cervical radiculopathy
4) Cervical myelopathy

Methods

The detailed description of the steps which we took is mentioned below and briefly in Table 2.

**Table 2:** Brief description of precautionary steps PPE: Personal protective equipment; TOCC: Travel to regions with a high prevalence of COVID-19, occupation with a high risk of COVID-19 infection, contact with people known to be infected with COVID-19, or proximity with a COVID-19 positive case.

Do’s	Dont’s
1.Preoperative period	
A) History, examination and screening Questinnare related to COVID 19 (Table 3)	A) Entry to visitors
B) TOCC information (Table 3)	B) Allow unnecessary attendant
C) Explain the hospital’s protocol for COVID-19 Infection	C) Overcrowing of patients in the ward and recovery room
D) Compulsory Mask for patient and attendant.	
2.Intraoperative period	
A) Proper donning and doffing technique of prescribed PPE	A)Avoid using High power tools
B) Limiting the number of people in the operating room	B) Unnecessary Crowd in operation room especially during intubation and extubation
C) Reducing door opening in the operating room	C) Cluttering of unnecessary equipment
D) Cautious use of electrocautery with suction	
E) Thorough cleaning of the operation room and all the equipment	
3. Postoperative period	
A) Mask for patient	A) Overcrowding in the recovery room
B) Social distancing in the recovery room	B) Keeping patient for longer time
C)Cleaning of bed and it's surface after the transfer of patient	C)Prolong stay in hospital
D) Immediate Postoperative round by Senior resident, Chief surgeon should be telephonically informed	D)Change in antibiotic protocols
E) Prescribed PPE by health care personnel	E) Interdepartmental Transfer for rehabilitation
F)Wound inspection and dressing – wear an N95 mask	F) A frequent follow-up visit to the hospital
G) Early rehabilitation and physiotherapy program	
H) Teleconsultation for follow up	

Precautions at admission

At presentation, history was recorded and clinical examination performed. The temperature and oxygen saturation at room air recorded for every patient. All patients were screened with a series of questions related to COVID-19 symptoms (fever, cough, fatigue, anorexia, shortness of breath, sputum production, loss of taste and smell, sore throat, diarrhea, nasal congestion) as well as TOCC information (travel to regions with a high prevalence of COVID-19, occupation with a high risk of COVID-19 infection, contact with people known to be infected with COVID-19, or proximity with a COVID-19 positive case) [8]. Information was also obtained regarding the previous infection and test for SARS-CoV-2 (Table 3).

**Table 3:** Coronavirus disease 2019 (COVID-19) screening questionnaire SARS-CoV-2: Severe acute respiratory syndrome coronavirus 2; RT-PCR: Reverse transcription-polymerase chain reaction

S.no			
1	Temperature		
2	Pulse Oximetry (Spo2)		
3	Travel History		
4	Occupation		
5	Contact with COVID positive Patient		
6	Close proximity to COVID 19 Positive patients		
7	Symptoms	Yes/ NO	If Yes, when was the onset of symptom
a	Fever		
b	Cough		
c	Fatigue		
d	Anorexia		
e	Shortness of breath		
f	Sputum production		
g	Loss of taste and smell		
h	Sore throat		
i	Diarrhea		
j	Nasal Congestion		
8)	Previous history of COVID -19 infection		
9)	Previous SARS- COV2 RT PCR test (Date)		Result – Positive/Negative

In the case of history or examination suggestive of COVID 19, the patient was isolated and tested. However, none of the emergency surgeries was delayed while awaiting test results. The sample was sent and surgery was conducted by donning full prescribed personal protective equipment (PPE).

Preoperative precautions

Preoperatively, patients and attendants explained about the precautions and measures (hand hygiene, wearing a mask, social distancing, restrictions of family members, and visitors in hospital) recommended by hospital authorities because of COVID-19 pandemic. The patients were provided with a surgical mask throughout the perioperative period. The patient was transferred to an operation theatre only on-call from the operation theatre to avoid overcrowding. The individual operating rooms have separate air-handling units with high-efficiency particulate air (HEPA) filters.

Precautions in the operation theatre

Intraoperatively, the anesthesia team consisted of a consultant, senior resident, junior resident, and an anesthesia technician. All preparation for general anesthesia was performed by the anesthesia team before shifting the patient to the operation table, during intubation either the consultant or the senior resident intubated the patient donning the prescribed PPE which consisted of Association for the Advancement of Medical Instrumentation (AAMI) level III gown, hood, N95 mask, face shield, boot covers, and gloves. The junior resident or the anesthesia technician was available to assist during the procedure [8]. During intubation and extubation, the surgical team was outside the operating room to limit the number of persons inside and minimize the risk of disease transmission. After intubation, the patient was handed over to the surgical team after about 20 minutes. The surgical team consisted of the chief surgeon, senior resident, junior resident, and scrub nurse. The entire surgical team was on hydroxychloroquine (HCQ) prophylaxis as advised by the Indian council of medical research (ICMR) [9]. The entire surgical team wore the prescribed PPE - N95 mask, hood with a face shield that wraps around the face and has an extension that can be placed inside the gown, AAMI level III gown, boot cover, and gloves [8,10,11]. The surgical knife was used for dissection and electrocautery was minimally used with suction wherever required to minimize aerosol generation. Kerrison rongeur, nibblers, and osteotomes were used in place of burr or harmonic scalpel which decreased the aerosol generation [12].

Wound closure was done over the drain with staplers and the occlusive dressing was applied.

During extubation, theatre personnel was limited to the anesthesia team and the patient was allowed to recover in the operating room to minimize the shifting to the recovery room. In case there was any other emergency case waiting for surgery the patient was shifted to the recovery room [13].

For the cases performed under local anesthesia like a transpedicular biopsy, the team inside the operating room was limited to surgeon, scrub nurse, and radiology technician to operate a C-arm image intensifier. After the procedure, the patient directly shifted to the ward.

As per institutional policy, following the procedure. the surfaces of all equipment in the operating room (including computers, lead gowns, case carts, operation chair, and so on) were thoroughly wiped down after each case. The surgeon and the surgical team changed scrubs after each case. Respirators (N95) were not discarded after each use unless soiled. A new respirator is required each day. As per institutional policy, the N95 masks were reused up to five times unless visibly soiled.

Precautions in the recovery room

In the recovery room, patients were separated from each other by a minimum distance of two meters (six-feet). Personnel taking care of the patient wore an N-95 mask as the patient is under the effect of general anesthesia, might not have control over cough and sneeze. However, the patient’s stay in the recovery area was minimized while maintaining perioperative safety. Patients were made to wear a triple-layer surgical mask in the postoperative period. Over-crowding and non-essential personnel traffic were restricted.

Post-operative care protocols

Postoperative rounds were done by the senior resident and informed to the chief surgeon telephonically. The surgical wound was inspected by the resident wearing an N95 mask on the third postoperative day and the drain, if any, was removed. The patient was usually discharged on the same day if the wound was healthy after teaching physiotherapy and other precautions to the patient and attendant to minimize the hospital-stay. For procedures that were performed under local anesthesia, the patients were discharged the next morning. Suture removal was advised to be done at a local hospital. Institute telemedicine number and the resident’s contact number was provided to the patient to consult in case of any surgery-related problem and to schedule follow up.

## Results

The present study included 13 patients (nine males and four females) with an average age of 35.4 years (range- 42-63 years).

Eight patients (61.5%) presented with SCI due to vertebral fracture with fall from height (87.5%) as the most common etiology. Road traffic accidents accounted for only one patient as the cause of SCI. Among the traumatic SCI patients, six (75%) were managed surgically with posterior decompression and instrumented fusion with pedicle screws while two patients (25%) were managed conservatively. There were four patients (30.8%) of tuberculosis of the spine of whom two (50%) were managed with posterior decompression, debridement, and stabilization with pedicle screws, samples for culture, biopsy, and CBNAAT were collected during the procedure; for the remaining two patients (50%), the trans-pedicular biopsy was performed to confirm the diagnosis for initiation of anti-tubercular therapy. Prolapsed intervertebral disc causing cauda equina syndrome was the reason for emergency surgery in one patient (7.7%). 

COVID-19 (SARS-CoV-2 RT-PCR) test was done in four patients (30.8%), in whom the most common symptom was fever (two patients (50%)) and all these patients were residents of high prevalence area for COVID-19 infection. Sore throat (25%), fatigue (25%), and low oxygen saturation (25%) were present in one patient each patient which prompted us to get the COVID-19 test. All patients were reported negative for COVID-19. Among the traumatic SCI patients, four were operated within 48 hours of injury as they were referred from other centers after primary care. All patients were managed within 24 hours of admission. The average hospital stay was 2.7 days (range four to five days) (Table 4).

**Table 4:** Patient demographics and results RTA: Road traffic accident; PF: Pedicular fixation

Sno.	Age/sex	Mode of trauma	Diagnosis	ASIA Score	TLICS Score	Surgery	COVID testing	Indication for COVID test	Result	Time interval	Hospital Stay
1	21/M	RTA	Burst # L1	A	7	Decompression and PF D12-L2	No	NA	NA	24 hrs	3 days
2	40/M	Fall	#D8	E	2	Conservative	No	NA	NA	Na	1 days
3	50/M	Fall	#D11	A	7	Decompression and PF D10 to D12	Yes	1)residence at High prevalence area 2)Spo2 – 88% on room air	Negative	48 hrs	3 day
4	30/M	fall	# L1	A	7	Decompression and PF D12to L2	Yes	1) Residence at High prevalence area	Negative	48 hrs	3 days
5	25/M	Fall	# L1	A	7	Decompression and PF D12to L2	NO	NA	NA	24 hrs	3 days
6	22/F	Fall	# L2	E	2	Conservative	No	NA	NA	NA	1 day
7	63/F	Fall	# L1	D	8	Decompression and PF D10 to L3	No	NA	NA	48hrs	3 days
8	43/M	Fall	#Dislocation D6/D7	A	7	Decompression and PF D5to D8	No	NA	NA	48 hrs	3 days
9	42/M	Infective pathology	Tuberculosis of spine from D8,D9,D12,L1,L2,L3 with paravertebral abscess	E	NA	Transpedicular Biopsy from D12 vertebra	No	NA	NA	24 hrs	1 day
10	33/F	Infective apthology	Tuberculosis of spine L4-L5 with paravertbral abscess	E	NA	Transpedicular Biopsy from L5 vertebra	No	NA	NA	24 hrs	1 day
11	25/M	Infective apthology	Tuberculosis of spine L2 to L3 with paravertebral abscess	C	NA	Decompression and PF D12to L5	Yes	1) Residence at High prevalence area 2)Fever 3) Fatigue	Negative	24 hrs	5 days
12	42/M	Infective apthology	Tuberculosis of spine L4-L5 vertebrae with paravertebral abscess	D	NA	Decompression and PF from L3 to S1	Yes	1) Residence at High prevalence area 2)Fever 3)Sore throat	Negative	24 days	5 days
13	24/F	Cauda Equina Syndrome	Proloapsed intervertebral Disc L4-L5	B	NA	Discectomy L4-L5	NO	NA	NA	24 hrs	3 days

## Discussion

Covid-19 crisis has exhausted the capacity of national health services even in developed countries. Most of the medical resources have been channeled for the management of this crisis and the elective services have been kept on hold. Still, there would be some unavoidable surgical conditions when a delay in surgery would significantly increase the morbidity and warrants consideration for appropriate intervention. Since there is a difference in the burden of disease, availability of resources, and the ability to surge capacity in a short span, the policy to deal with this crisis must be individualized to some extent. We tried to develop a standard operating procedure for the management of patients needing emergent/urgent spine surgery taking input for the recent evidence.

The most common mode of injury in vertebral fractures is a motor vehicle accident [13,14]. However, in our study, fall from height (87.5%) was the commonest mode of trauma. The possible explanation is that due to the nationwide lockdown, there was a significant reduction in surface transportation and people were generally refraining from traveling for non-essential work. Due to the limited capacity for SARS-Cov-2 RT-PCR testing and the centers performing COVID tests, comprehensive screening history, and physical examination becomes important to triage the patient for the test. History should focus on the symptoms of COVID-19, travel to high prevalence area, or any contact with a known patient. It is also important to enquire whether the patient had been infected with COVID-19 earlier or ever been tested as there are reports of individuals who got infected with COVID-19, recovered, and then again tested positive [14]. Secondly, it will also help in assessing if the patient might be in the incubation period. However, in case of an emergency, the surgeries should not be delayed. A sample can be sent for RT-PCR and surgery should be performed donning proper PPE without waiting for the result. As asymptomatic patients can transmit the infection it becomes prudent to assume that all patients requiring orthopedic intervention as COVID-19 positive and they should be managed accordingly from their admission into the hospital to their exit from the hospital during their discharge [1].

Trans-pedicular screw fixation is one of the main modalities of spinal instrumentation today and patients are stabilized with pedicle screws and rods [15].

Patient and attendant education regarding the administrative policies to prevent contracting/transmitting infection while in hospital as well as education regarding wearing a mask, hand hygiene, social distancing serves the purpose of making patients aware of their safety [4,7,10].

In the operation theatre complex, it is mandatory to avoid overcrowding in all areas (patient waiting area, operating room, and the recovery room). Inside operation theatre it is important to limit the number of staff allowed as SARS-CoV-2 is mainly transmitted via aerosolized droplets, so by decreasing the air turbulence and the number of air particles decreases the disease transmission; it will help in implementing the social distancing concept while also decreasing the demand for PPE [8,10,11,16-18].

To decrease the risk of transmission from airborne droplets it is mandatory to use PPE. The surgical team must wear PPE which includes AAMI III gowns, gloves, face masks, and N95 with face shield/goggles to minimize the risk of transmission and cross-infection [8,11,12]. Association for the Advancement of Medical Instrumentation (AAMI) ratings are based on the level of fluid protection in the critical zone or chest region of the surgical gown. Surgical gowns AAMI-level-III (typically those found in operating rooms) are recommended for use during surgical and aerosolized blood-generating procedures [8,11,12,17-19].

At our institute, N-95 masks are reused unless they are visibly soiled. Each HCW is given a set of five N-95 masks that are numbered from 1 to 5 and HCWs are supposed to write their name and number over it. After the duty shift, HCWs are supposed to put it in sealable plastic cover and next day use the next mask. The masks are deposited at the designated place from where the infection control nurse (ICN) collects them for drying under the sun on two consecutive days for at least nine hours/day. The masks are issued to the same user only after the fifth day of submission. Each mask is used maximum for five times. Subsequently, the mask is disposed of appropriately. Single-use of the N-95 mask is recommended if it is contaminated with blood, respiratory or nasal secretions, or other bodily fluids from patients. It should be discarded following close contact with or exit from, the care area of any patient co-infected with an infectious disease requiring contact precautions, and if it is damaged or clogged. The rationale behind this is dry heat 70^o^C exposure for 60 minutes as an accepted method for decontaminating the N-95 masks. Further, it is documented that the COVID-19 virus does not survive on inanimate surfaces beyond a period of five days [20,21]. 

As per the advisory issued by the Ministry of health and family welfare on re-processing and re-use of eye-protection (goggles), the goggles are to be reused; after each use, the goggles are cleaned with soap and water and then immersed in freshly prepared 1% sodium hypochlorite for ten minutes. This is followed by cleaning with water and then allowed to air dry completely and then store in clean cover. The goggles are to be discarded if broken or rendered optically non-clear on repeated usage. The recommended maximum reuse is for up to six times [22]. These methods devised to reuse N-95 and goggles are very helpful in the judicious use of limited resources without increasing the risk of infection. The proper and meticulous technique of donning and doffing should be practiced each time to prevent infection [23].

Our surgical team was on HCQ prophylaxis as recommended by the ICMR in a dose of 400 mg twice a day on day one and 400 mg once a week for seven days [9]. HCQ has antiviral efficacy and has shown a reduction of infectivity/log reduction in viral RNA copy of SARs-CoV2 and has a low incidence of infection to those who are taking it. The drug is contraindicated in persons with a known case of retinopathy, hypersensitivity to HCQ or 4-aminoquinoline compounds, glucose-6-phosphate dehydrogenase (G6PD) deficiency, and pre-existing cardiomyopathy and cardiac rhythm disorders. 

It is not recommended for prophylaxis in children under 15 years of age and during pregnancy and lactation [24]. 

The aerosols generating procedures in orthopedic surgery include the use of electrocautery, high-speed burr, ultrasonic devices, and power tools like an oscillating saw, drills, reamers, and pulse lavage [12]. While it is unknown if the COVID-19 particles can survive electrocautery, minimizing the amount of smoke in the operating room helps in decreasing potential transmission. Similarly, it is unknown that a virus can spread through body fluids, soft tissue, or bone particulate, it is wise to avoid harmonic scalpels and high-speed burrs [8,11,12,18].

The postoperative length of stay in the hospital should be minimized. The post-operative care (like dressing, intravenous antibiotics, etc.) can be managed at the patient’s home or the nearest hospital. The patient is instructed to perform self-directed physical therapy at home and transfer to inpatient rehabilitation should be minimized which can significantly reduce the length of hospital stay [10,11,16,17,19,25]. Before the COVID-19 pandemic, we were transferring the paraplegic patients to our hospital-based rehabilitation center where discharge time was about six weeks. During this pandemic, we started teaching physiotherapy and care to attendants from day one which decreased the hospital stay to 3.7 days. 

Post-discharge visits to the hospital have been minimized with the majority of the follow-up by the surgical team through telemedicine or remote consultations (e.g. telephone or video consultation). The hospital visits were limited to those who are having issues/complications such as wound healing problems, suspected fracture, stiffness, and non-compliance to physiotherapy [4,7,11-13].

There are chances of a “second wave” which were seen in SARS and Spanish flu which may lead to a sudden increase in COVID-19 cases [10]. We need to be vigilant and be prepared for it. We should strictly adhere to infection control policy, use masks, and practice social distancing. 

The above structural organization and management protocol helped us to provide efficient care to emergency spine conditions, without undue stretching the resources of a tertiary care center which is also catering to a large number of COVID-19 patients. An attempt is made to reduce infection transmission to health care workers without compromising the patient's safety.

We recognize that the small patient number is a limitation of this study and follow up is short. We have not assessed the problem faced by a home-based rehabilitation program and is a subject of further research

## Conclusions

In these unusual times of the COVID-19 pandemic, the healthcare institutions must adopt the emerging pieces of evidence on the safety of HCW’s into the existing hospital policies. Usual theatre practices need to be modified to reduce the risk of virus transmission during surgical procedures to the operation theatre team. The protocol we described has enabled our surgical team to work efficiently and safely without compromising on patient care.
